# Effects of Bench Press Volume on Performance, Recovery, and Physiological Response

**DOI:** 10.3390/sports14020076

**Published:** 2026-02-09

**Authors:** José A. Páez-Maldonado, África Calvo Lluch, Manuel Ortega-Becerra, Fernando Pareja-Blanco

**Affiliations:** 1 Center Attached to the University of Seville, University of Osuna, 41640 Osuna, Spain; 2 Science-Based Training Research Group, Physical Performance & Sports Research Center (CIRFD), Universidad Pablo de Olavide, 41013 Seville, Spain; 3 Faculty of Sports Sciences, Department of Sports and Computer Sciences, Universidad Pablo de Olavide, 41013 Seville, Spain

**Keywords:** rate of recovery, lactate, training volume, blood pressure response, oxygen saturation, velocity-based training

## Abstract

Background: The purpose of the study was to examine the effects of training volume in bench press (BP) on acute mechanical, metabolic, and cardiovascular responses, and the time course of recovery. Methods: Fourteen men with moderate resistance training experience performed, in randomized order and separated by one week, three BP protocols differing in volume: 3 (LOW), 15 (MOD), and 24 (HIG) repetitions. To isolate the effect of training volume by minimizing fatigue accumulation across repetitions, short rest periods were inserted between repetitions. The rest duration was individualized based on the performance impairment induced in each repetition. A battery of tests was performed at baseline (Pre) and post-exercise, in the following order: (a) heart rate (HR), blood systolic and diastolic pressure (SBP and DBP), and oxygen saturation (SpO2), (b) blood lactate, and (c) dynamic strength test, which was also conducted at 24 h-Post and 48 h-Post. Results: Performance within-session (best, average, and last velocity, as well as velocity loss) was similar for all protocols. A significant “protocol × time” interaction was observed for SBP, although no significant differences between protocols were found. No significant differences were observed for DBP or SpO2. All protocols showed similar lactate concentrations at Post and similarly increased velocity at 60% 1RM load at 24 h-Post and 48 h-Post. Conclusions: individualizing inter-repetition rest periods based on velocity loss allows matching fatigue across different bench press volumes, which produced similar mechanical, metabolic, and cardiovascular responses, indicating that volume alone does not determine acute physiological load.

## 1. Introduction

The design of a resistance training (RT) program is a complex task that requires careful supervision and manipulation of several variables that constitute the training stimulus. These variables include intensity, volume, execution velocity, rest periods, type and order of exercises, training frequency, and recovery time [1]. Combinations of these variables directly affect mechanical, metabolic, and cardiovascular responses, as well as long-term adaptations [2]. Therefore, understanding the mechanical and physiological aspects underlying different resistance exercise protocols (REPs) is crucial for elucidating the mechanisms that maximize performance [3].

Proximity to failure is a variable that influences training stimuli [4]. It has been shown that proximity to failure impacts the acute mechanical and metabolic responses [5] and requires longer recovery periods [6,7,8]. Understanding the time needed to recover baseline values after RT is crucial, as it may hinder the development of other physical, technical, or tactical components [9]. If there is an imbalance between overload and recovery, undesirable adaptations may occur [10]. Therefore, it is essential to consider rest periods between sessions to promote optimal recovery.

To reduce fatigue during high-volume training, adding short rest periods between repetitions can help prevent performance declines and shorten recovery time afterward [11,12]. It has previously been demonstrated that incorporating rest periods of 10–20 s between repetitions (i.e., cluster set) is sufficient to reduce mechanical and metabolic fatigue during training sessions compared to continuous repetitions [13].

Heart rate (HR) has been widely employed as an indicator of the physiological regulation system’s response to physical effort [14,15,16]. Performing consecutive repetitions within a set is associated with a gradual elevation in HR and blood pressure [17,18]. Although the systolic blood pressure (SBP) of the first repetition is higher with higher intensities [19], it has been suggested that volume is the main factor influencing SBP [20,21,22]. In this regard, it has been observed that lower-intensity sets with more repetitions elicit greater cardiovascular responses than higher-intensity sets with fewer repetitions [20,21,22]. Likewise, previous research has shown that incorporating short rest periods between repetitions is an effective strategy to reduce SBP increments during RT [23,24].

Several studies have explored the acute effects of different training volumes [6,8], but none have controlled for the possible influence of fatigue linked to a specific volume. To attribute training effects specifically to volume rather than to fatigue accumulated within a set, it is essential to design studies that compare different training volumes while keeping fatigue levels consistent. One way to control this factor is to monitor lifting velocity on each repetition. To reduce the velocity loss (VL) associated with higher volumes, brief rest periods within a set (i.e., inter-repetition rests) can be implemented [11,12]. Additionally, these recovery periods could be individualized according to performance in each repetition, with the aim of sustaining performance (i.e., maintaining lifting velocity) as effectively as possible. Thus, the objective of the present study is to examine the acute effects of three distinct bench press (BP) training volumes, isolating the effect of volume by introducing individualized rests after each repetition, on mechanical, metabolic, and cardiovascular responses, as well as their subsequent recovery time course. We hypothesized that the three REPs would elicit similar mechanical, metabolic, and cardiovascular responses, but that the higher-volume protocol would require longer recovery time.

## 2. Materials and Methods

### 2.1. Subjects

Fourteen moderately resistance-trained men (age 23.4 ± 4.4 years; height 1.73 ± 0.08 m; body mass 70.9 ± 9.5 kg; relative one-repetition maximum [1RM] per body mass = 0.90 ± 0.20), with a training history ranging from 1 to 2 years, participated in this study. All participants were provided with detailed information about the study’s purpose and procedures and signed informed consent forms. Participants with a history of cardiovascular disease, hypertension, or any cardiac-related condition were excluded from the study. The study was approved by the Research Ethics Committee (Ref: 03-819, 11 October 2019) in accordance with the Declaration of Helsinki.

### 2.2. Experimental Design

A randomized, within-participant, crossover design was used to investigate the acute mechanical, metabolic, and cardiovascular responses to three BP protocols that differed in total volume: LOW (only 3 repetitions), MOD (15 repetitions), and HIG (24 repetitions). The protocols were performed weekly for 3 weeks, with at least 7 days between sessions. All protocols used the same relative intensity (70% 1RM). Each session consisted of a set with short rest periods between repetitions to minimize fatigue accumulation. When the difference in mean propulsive velocity (MPV) between the fastest repetition and the following ones was lower than 0.03 m·s^−1^, the rest period was 10 s. If the VL ranged from 0.04 to 0.06 m·s^−1^, the rest period between repetitions was extended to 20 s. Every increase of 0.03 m·s^−1^ in VL corresponded to a 10 s increase in the rest period. This was done to avoid fatigue accumulation and maintain MPV across repetitions. A battery of tests at baseline (Pre) and post-intervention was conducted for each protocol in this order: (a) cardiovascular assessment, (b) blood lactate, and (c) dynamic strength test. Additionally, to examine the time course of recovery, the dynamic strength test was performed at 24 h-Post and 48 h-Post. One week earlier, a progressive loading BP test was conducted to establish the individual load-velocity relationship for each subject. Participants were instructed to maintain their usual diet, abstain from caffeine for 12 h before attending the study, and avoid any other strenuous physical activity during the 72 h preceding each protocol. Protocols were conducted in a research laboratory under the direct supervision of researchers, with the same environmental conditions (20 °C and 60% humidity), and at the same time of day (±1 h). Participants received strong verbal encouragement to exert their maximum effort during all protocols.

### 2.3. Testing Procedures

#### 2.3.1. Progressive Loading Test

A linear velocity transducer (T-Force System, Ergotech, Murcia, Spain) was utilized to assess individual load-velocity relationships and 1RM load in the BP exercise, whose reliability has been reported [5,25]. Participants were positioned supine, with full back support and their feet on the bench. They maintained a pronated grip at ~150% of the biacromial width throughout all protocols. The eccentric phase of the movement was performed at controlled velocity, and participants held the bar on their chest for approximately 1 s to minimize the rebound effect and achieve more consistent measurements [26]. During the concentric phase, subjects were instructed to lift the bar with maximal velocity and effort upon receiving the command. It was not allowed to throw the bar at the end of the concentric phase. The velocity variable used in this study was the MPV, defined as the concentric movement in which the measured acceleration exceeds the acceleration due to gravity (−9.81 m·s^−2^) [27]. The warm-up consisted of one set of 6 BP repetitions with 20 kg. The initial load was 20 kg and was progressively increased by 10 kg (or 5 kg when MPV was <0.40 m·s^−1^) until the MPV reached ≤0.30 m·s^−1^. Each subject completed a total of 5.1 ± 0.9 increasing loads. Participants performed three repetitions with light loads (>0.80 m·s^−1^), two repetitions with medium (0.80–0.60 m·s^−1^), and only one with heavy loads (<0.60 m·s^−1^). Inter-set recovery periods were 3 min. The best repetition for each load was considered for subsequent analysis. Individualized linear load-velocity relationships were obtained from this test. For 1RM strength estimation, the absolute load that each subject could lift at 0.18 m·s^−1^ was calculated [27].

#### 2.3.2. Cardiovascular Assessment

During all measurements, the participants were instructed to hold their seats with the back well supported against the chair backrest. The legs should be touching the floor, not crossed, and the hand should be relaxed, not gripping tightly, and in a resting position. The left arm should be resting at about heart level, hand relaxed. The sphygmomanometer must be in contact with the skin. During cuff inflation, participants were not allowed to talk. To ensure accurate measurement of oxygen saturation (SpO2), the designated pulse oximeter (Edan Instruments, Inc., Shenzhen, China) placement area (either the third or fourth finger of the right hand) was thoroughly cleaned with hydrophilic, alcohol-soaked cotton. After 1 min of auto-calibration, oximetry data recording commenced. SBP, diastolic blood pressure (DBP), HR, and SpO2 measurements were conducted in the baseline state, after warm-up, and post-exercise with Edan M3A Vital Signs Monitor (Edan Instruments, Inc., Shenzhen, China) 

#### 2.3.3. Blood Lactate Concentration

The finger was cleaned with alcohol and dried with cotton. A slight puncture was made on the fingertip to obtain the first drop of blood, which was discarded. Next, the second drop of blood was applied to the test strip. Lactate levels were measured using a portable lactate analyzer (Lactate Pro 2, Arkray, Kyoto, Japan). This system has demonstrated high reliability within a physiological range of 0.5–25.0 mmol·L^−1^ [28].

#### 2.3.4. Dynamic Strength Test

This test consisted of performing 3 BP repetitions at 60% 1RM. The BP execution technique and settings were the same as described in the “progressive loading test” section. The highest MPV achieved during the three repetitions was recorded for further analysis.

#### 2.3.5. Resistance Exercise Protocols

**Figure 1:**
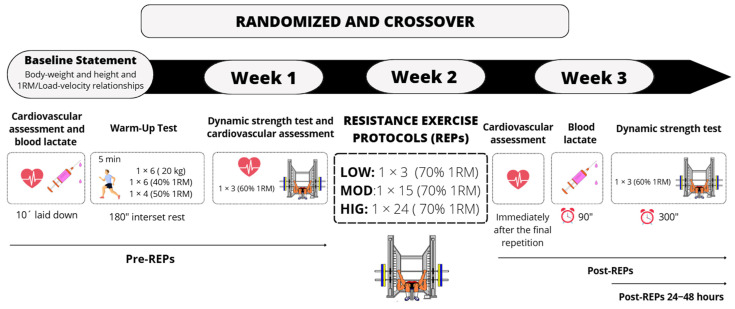
Schematic representation of the different resistance exercise protocols (REPs) and the battery of tests performed before and after each REP, and the timeline. LOW: the low-volume REP; MOD: the moderate-volume REP; HIG: the high-volume REP; %1RM: percentage of one-repetition maximum. PRE: before the REPs; POST: after the REPs.

The protocols are depicted in Figure 1. All subjects were required to lie down for 10 min before baseline data acquisition to minimize the effects of previous activity. Subsequently, baseline measurements were taken for cardiovascular variables and blood lactate. Then, a standardized BP warm-up was performed, consisting of 5 min of jogging, 6-6-4 repetitions with 20 kg, 40%, and 50% 1RM, respectively, with a 3 min rest between sets. The BP execution technique and settings were consistent with those outlined in the “progressive loading test” section. Relative loads were determined from the individual linear load-velocity relationship (R^2^ = 0.998 ± 0.001) derived from the progressive loading test. After the warm-up, the dynamic strength test (3 repetitions at 60% 1RM) was performed, serving as a baseline strength measure. The absolute loads (in kg) were individually adjusted to ensure the corresponding MPV matched (±0.03 m·s^−1^) the prescribed %1RM for each session. We used a range of 0.03 m·s^−1^ because this is the smallest detectable change in MPV under the setting employed in the present study [25]. Then, the corresponding protocol was carried out: LOW, MOD, and HIG performed 3, 15, and 24 repetitions, respectively, at 70% 1RM. To minimize fatigue accumulation during the protocols, short inter-repetition rest periods (10 s) were added. Every increase of 0.03 m·s^−1^ in VL (i.e., difference in the MPV between the fastest repetition and the successive repetitions) corresponded to a 10 s increase in the rest period. Therefore, if the VL ranged 0.04–0.06 m·s^−1^, a 20 s rest period was imposed; if the VL was 0.07–0.09 m·s^−1^, 30 s, and so on. After the last repetition, the battery of tests was repeated at Post as follows: (1) cardiovascular assessments (immediately after finishing the last repetition); (2) blood lactate (at 90 s); and (3) dynamic strength test (at 300 s). Also, the dynamic strength test was assessed at 24 and 48 h post-exercise.

### 2.4. Statistical Analyses

Values are expressed as mean ± standard deviation (SD). Normality was verified using the Shapiro–Wilk test. A one-way repeated-measures ANOVA was performed to compare performance during protocols. A two-way repeated-measures ANOVA with a 3 (protocol) × 2 (time) design was conducted to analyze the lactate responses to each REP. Additionally, two-way repeated-measures ANOVAs with 3 (protocols) × 4 (time) and 3 (protocols) × 3 (time) designs were used to analyze post-exercise recovery and cardiovascular responses, respectively. For pairwise comparisons, Bonferroni adjustments were applied. Significance was accepted at *p* ≤ 0.05. Pre-post effect size (ES) values were calculated using Hedges’ g based on the pooled SD [29]. All statistical analyses were performed using SPSS software version 25.0 (SPSS, Inc., Chicago, IL, USA) and Microsoft Excel 2007 for ES calculations. Figures were designed using GraphPad Prism 5 (GraphPad Software, San Diego, CA, USA) and SigmaPlot 12.0 (Systat Software Inc., San Jose, CA, USA).

## 3. Results

### 3.1. Descriptive Characteristics of the Resistance Exercise Protocols

Table 1 shows the mechanical characteristics of each protocol. No significant differences were found in the relative intensity (~70% 1RM) and absolute load (~45 kg) employed in each protocol (both *p* > 0.05). All protocols achieved a similar MPV values (best: ~0.57 m·s^−1^, average: ~0.52 m·s^−1^; last: ~0.51 m·s^−1^, all *p* > 0.05). The VL was also similar for the 3 protocols (*p* = 0.44).

**Figure 2:**
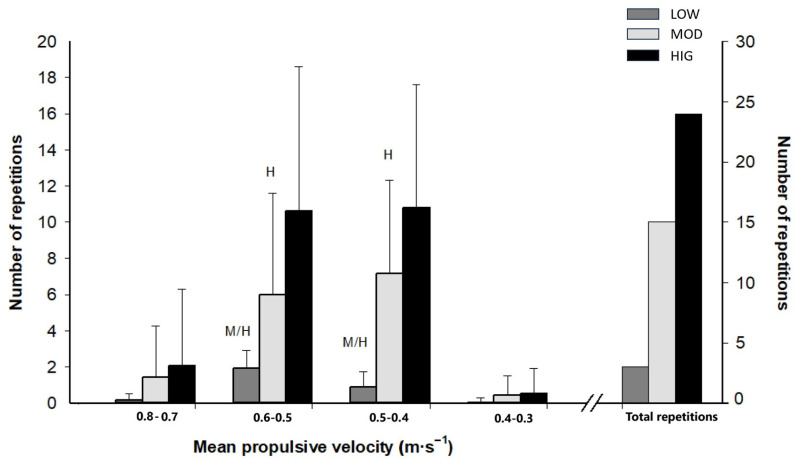
Number of repetitions performed in each velocity range, and total number of repetitions completed in each protocol. Data are mean ± standard deviation, *n* = 14. LOW: the low-volume protocol; MOD: the moderate-volume protocol; HIG: the high-volume protocol. Statistically significant differences with MOD: ^M^ *p* ≤ 0.05. Statistically significant differences with HIG: ^H^ *p* ≤ 0.05.

Figure 2 shows the number of repetitions performed within each velocity range, as well as the total number of repetitions completed for each protocol. At 0.6-0.5 and 0.5-0.4 m·s^−1^ ranges, HIG accumulated more repetitions than MOD and LOW, and MOD completed more repetitions than LOW. Table 2 presents the percentage of repetitions performed within each velocity interval. No significant differences were observed between protocols when the number of repetitions was expressed as a relative measure.

### 3.2. Cardiovascular Assessments

**Figure 3:**
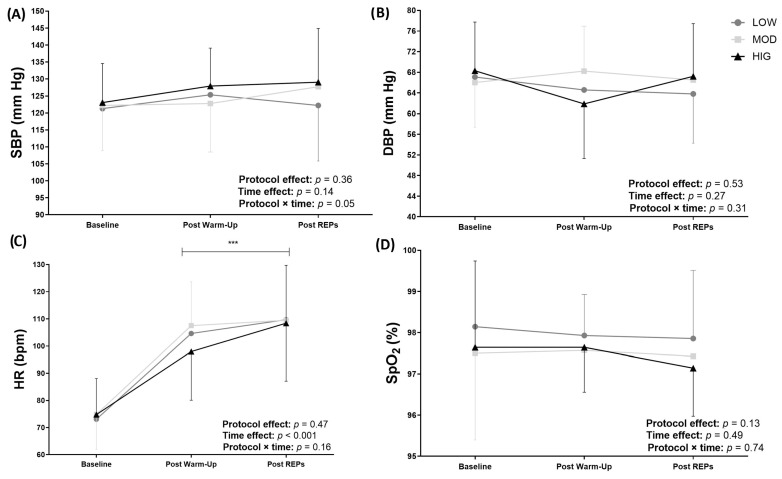
(**A**) Systolic blood pressure (SBP), (**B**) diastolic blood pressure (DBP), (**C**) heart rate (HR), and (**D**) saturation oxygen (SpO2). Basal: before warm-up; post-warm-up: after warm-up; and Post REPs: after the resistance exercise protocol. Data are mean ± standard deviation, *n* = 14. LOW: the low-volume protocol; MOD: the moderate-volume protocol; HIG: the high-volume protocol. Intra-protocol significant differences from baseline: *** *p* < 0.001.

Figure 3 shows the acute cardiovascular response to all protocols. A significant “time effect” (*p* < 0.001) was observed for HR, without differences between protocols. A significant “protocol × time” interaction was observed for SBP (*p* = 0.047); however, no significant differences between protocols were found. DBP and SpO2 remained unchanged for all protocols.

### 3.3. Blood Lactate Concentration

**Figure 4:**
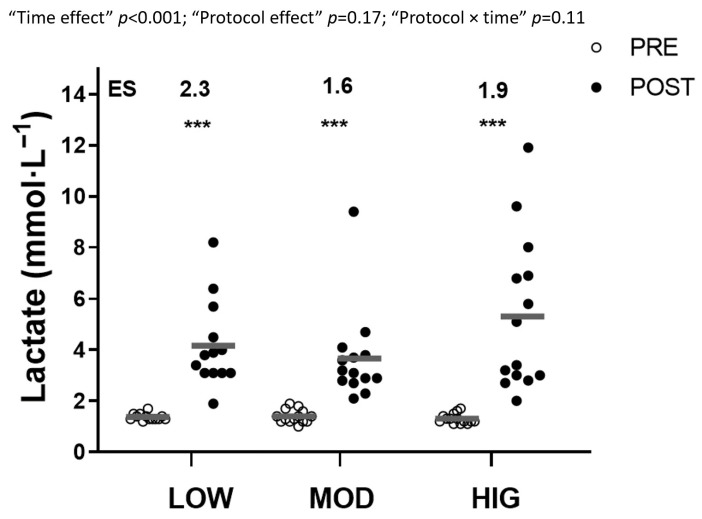
Lactate concentration before and after each protocol. Data are presented as individual responses (*n* = 14). LOW: the low-volume protocol; MOD: the moderate-volume protocol; HIG: the high-volume protocol; ES: Effect size within the protocol from Pre to Post. Statistically significant within-protocol differences compared to Pre: *** *p* < 0.001.

Figure 4 displays the acute responses of blood lactate to each protocol. A significant “time effect” (*p* < 0.001) was found, with no significant “protocol × time” interactions. All protocols showed an increase in post-exercise lactate (ES ranged from 1.6 to 2.3).

## 4. Dynamic Strength Test

**Figure 5:**
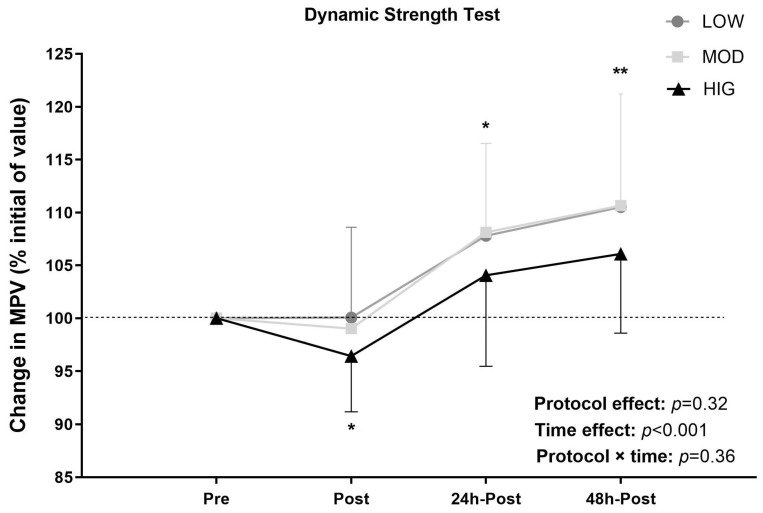
Changes in the velocity attained against the 60% one-repetition maximum (1RM) load for each protocol at the different time points. Data are presented as mean ± standard deviation (*n* = 14). LOW: the low-volume protocol; MOD: the moderate-volume protocol; HIG: the high-volume protocol. Statistically significant within-protocol differences with Pre: * *p* < 0.05, ** *p* < 0.01.

**Figure 6:**
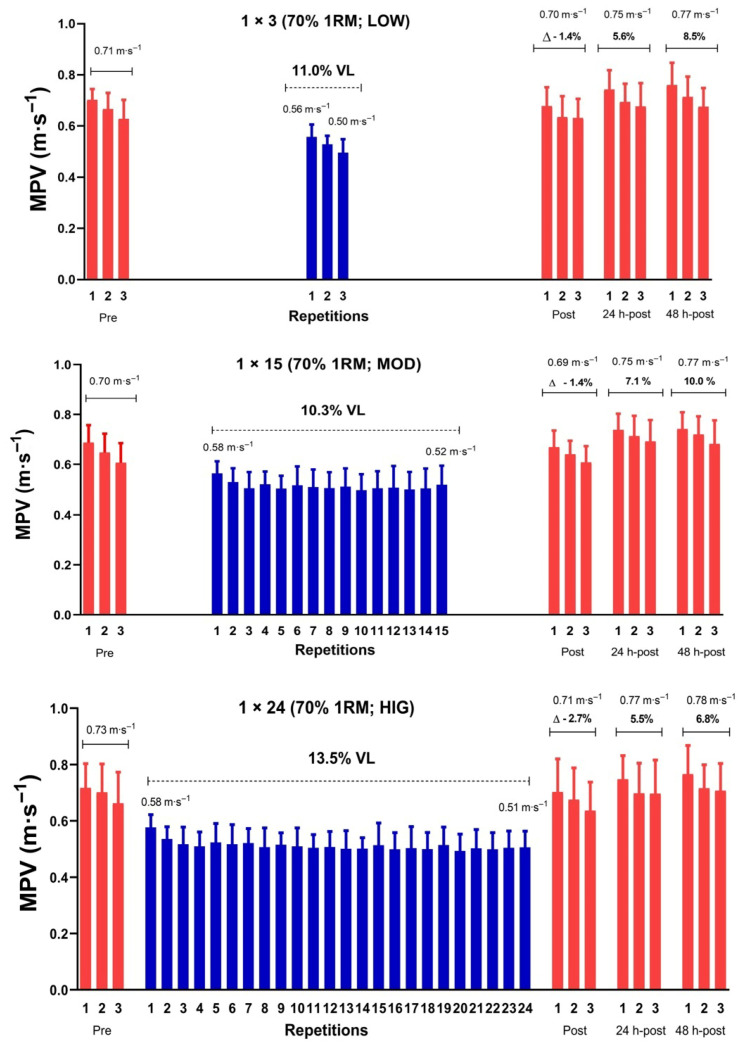
Example of monitoring the mean propulsive velocity (MPV) during each protocol and quantification of the relative change in MPV against the velocity at 60% 1RM load at different time points after each resistance exercise protocol. Data are presented as mean ± standard deviation (*n* = 14). LOW: the low-volume protocol; MOD: the moderate-volume protocol; HIG: the high-volume protocol; VL: magnitude of velocity loss expressed as relative difference between the best and the last MPV within protocol; 1RM: one-repetition maximum; Δ: relative change compared to Pre.

Figure 5 shows the evolution of BP performance at Pre, Post, 24, and 48 h post-exercise, assessed through the MPV achieved at 60% 1RM. A significant “time effect” (*p* < 0.001) was observed, without significant “protocol × time” interactions. HIG showed a significant deterioration at post-exercise. LOW and MOD showed significant improvements at 24 and 48 h post-exercise. Figure 6 summarizes the evolution of BP performance across protocols and at different time points up to 48 h post-exercise.

## 5. Discussion

To the best of our knowledge, this is the first study to individualize recovery time between repetitions based on performance loss in each repetition, thereby minimizing the influence of fatigue on acute mechanical, metabolic, and cardiovascular responses to BP training volume. The main findings indicate that, when fatigue levels are equalized during the set, different BP training volumes do not result in differences in the mechanical, metabolic, or cardiovascular responses induced. These findings demonstrate that this design could independently examine the effect of volume while avoiding the potential influence of fatigue (understood as performance loss).

### 5.1. Performance During the Resistance Exercise Protocols

Similar performance (i.e., MPV values) was found across all protocols despite the different accumulated volumes (LOW: 3 repetitions; MOD: 15 repetitions; HIG: 24 repetitions). The findings support previous research reporting that introducing short rest periods between repetitions helps maintain stable lifting kinematics, thereby preserving performance during a training session [30,31,32,33,34]. The literature shows that short rest intervals enhance the recovery of bioenergetic factors that sustain energy reserves, such as adenosine triphosphate (ATP) and phosphocreatine (PCr) concentrations, and improve metabolite clearance, thereby maintaining better performance [35,36]. However, this is the first study to use an individualized rest length between repetitions based on the performance loss in each repetition. These findings highlight the importance of considering set configurations when designing RT programs to accumulate greater training volume while minimizing fatigue.

In contrast, traditional protocols (i.e., without rest between repetitions) are characterized by progressive VL as sets approach muscular failure, which is commonly interpreted as an inevitable consequence of higher volumes [8,37]. The present findings demonstrate that performance deterioration is not an inherent outcome of higher training volumes when fatigue accumulation is controlled, supporting the notion that VL—rather than volume per se—is the primary determinant of acute mechanical performance decline [37].

### 5.2. Cardiovascular Response

Research has shown that the blood pressure response is higher after 15RM than after 4RM during the leg extension exercise [38]. Additionally, when volume was equalized (four repetitions), blood pressure was similar for both loads (4RM vs. 15RM). However, when intensity was equalized (15RM), blood pressure increased with increasing training volume, particularly as muscular failure approached [38]. Therefore, the literature suggests that training volume, especially near muscle failure, may be more relevant than intensity in determining the acute blood pressure response. Moreover, it has been shown that training to muscular failure imposes greater cardiovascular stress (increased SBP) than sets with rest periods between repetitions or groups of repetitions [16,23,24].

In the present study, it was observed that SBP and DBP during the BP exercise at 70% 1RM with different volumes were similar. Therefore, incorporating short rest intervals between repetitions helps maintain stable blood pressure during training sessions, regardless of the volume performed. These findings suggest that incorporating short rest intervals between repetitions could be effective for reducing cardiovascular stress during training sessions, enabling higher-volume training without excessive cardiovascular strain. These results, along with those found in the literature, suggest that it is the fatigue produced during the training set, rather than the volume, that causes the increase in blood pressure.

The RT increases sympathetic activity, elevating HR to meet the increased blood demand in the active muscles [39]. However, in the present research, the HR showed no differences between protocols, despite the difference in the volumes performed. Another important finding of this study was the absence of a decrease in SpO2, indicating the availability of oxygen during training sessions, despite differences in accumulated volume across protocols. Likely, the introduction of short rest periods between repetitions prevents blood flow restriction caused by continuous contractions of the active muscles, which would otherwise lead to a decrease in SpO2. This further supports evidence that cardiovascular strain observed in high-volume resistance exercise is largely mediated by sustained contractions and fatigue accumulation rather than repetition number alone [16,23,24].

### 5.3. Blood Lactate Response

Regarding post-exercise lactate concentration, it did not increase with more repetitions. Previous studies have shown that lactate rises with higher volume [40,41]. However, the increase in lactate is attenuated when short rest intervals between repetitions are used, compared with traditional sets (i.e., continuous repetitions without rest), while matching training volume [13,16]. Our results may be attributed to the fact that short rest intervals reduce fatigue and decrease metabolic stress [13,42]. Additionally, it may allow partial replenishment of muscle PCr and ATP reserves [35,36], thereby attenuating increases in inosine monophosphate (IMP) [43] and preventing purine loss from muscle tissue [44]. In failure-based protocols, lactate accumulation is often interpreted as a direct consequence of higher volume; however, the present findings indicate that metabolic stress is more strongly associated with fatigue accumulation induced by continuous repetitions and proximity to failure [13,16,42].

Elevations in blood lactate lead to hydrogen ion (H+) accumulation, which can lower pH [45]. Type III and IV afferent fibers can transmit information to the central nervous system (CNS) about changes in pH and exercise-induced discomfort. As a result, severe plasma acidosis can impair exercise performance by reducing CNS signaling to the muscles [46]. The inclusion of different recovery periods, individualized based on the subject’s performance in each repetition, has allowed for equalizing the level of accumulated metabolic stress across protocols despite the considerable differences in the volume completed in each.

### 5.4. Time Course of Recovery

The acute effects of the protocols showed that during training sessions, a higher volume elicited mechanical responses similar to those elicited by lower and moderate volumes. These findings contrast with failure-based training models, in which higher volumes are typically associated with greater neuromuscular fatigue and slower recovery kinetics [37,44]. According to previous research, reaching muscular failure during repetitions significantly disrupts the muscle’s energy balance and leads to considerable depletion of muscle purines [44]. On the other hand, performing half of the maximum number of repetitions per set helps maintain cellular homeostasis [35,36]. Therefore, incorporating short rest periods between repetitions could help achieve high volume while minimizing neuromuscular fatigue.

Despite scientific evidence supporting the use of short intra-repetition rest intervals to maintain performance during training [11,47], little is known about how rest periods within each set influence the temporal course of recovery. Interestingly, the HIG protocol achieved a recovery rate similar to that of the LOW and MOD protocols, despite accumulating a higher training volume. Recently, Cornejo-Daza et al. (2024) [37] were the first to analyze the progression of recovery across four protocols that differed in VL within the set and set configuration (traditional set vs. cluster set). The main findings of this research were that protocols with higher VL led to more pronounced fatigue and slower recovery. Additionally, cluster sets allow for greater volume than protocols with similar VL and showed faster recovery than those with similar volume. Together with the present findings, this supports the concept that VL and fatigue accumulation—rather than total training volume—are key determinants of recovery time course following resistance exercise.

The present study is not without limitations. First, the exercise protocol was based on the BP exercise. Therefore, the results may differ when using other exercises (e.g., lower-body movements). Second, although participants were instructed to maintain their usual dietary habits and avoid any supplements that could interfere with the intervention, dietary intake was neither monitored nor quantified. Finally, the small sample size should be considered when interpreting the results, and the findings may not be generalizable to other populations, such as female participants or high-performance athletes.

## 6. Conclusions

This study is the first to individualize intra-repetition rest based on real-time performance loss, enabling the manipulation of training volume while minimizing fatigue during the BP exercise. When fatigue was equalized, different BP volumes produced similar mechanical, metabolic, and cardiovascular responses, indicating that volume alone did not determine the acute physiological load. Individualized short rest intervals preserved performance, attenuated cardiovascular and metabolic stress, and enabled higher volumes without impairing recovery. These findings highlight the importance of set configuration in RT and suggest that incorporating brief intra-repetition rest may allow trained males to accumulate greater training volume while minimizing fatigue. Future studies should examine whether these results extend to other exercises, populations, and long-term training adaptations.

## 7. Practical Applications

The use of individualized short rest intervals between repetitions allows athletes to accumulate higher training volumes while minimizing fatigue and preserving repetition velocity. This set configuration reduces cardiovascular and metabolic stress, making it a suitable option for individuals who may be sensitive to blood pressure elevations or excessive metabolic load. Because recovery was not impaired despite higher volumes, this approach may help coaches increase total weekly training volume without compromising subsequent sessions. Overall, individualized intra-repetition rest offers a practical strategy to enhance volume-oriented training while maintaining movement quality and reducing performance decline.

## Figures and Tables

**Table 1:** Descriptive characteristics of the scheduled and actually performed resistance training protocols.

**Acronym**	**LOW**	**MOD**	**HIG**	
**Scheduled**				
Set × repetitions	1 × 3	1 × 15	1 × 24	
Intensity (%1RM)	70%	70%	70%	
Target velocity (m·s^−1^)	0.56 ± 0.02	0.56 ± 0.02	0.56 ± 0.02	
**Actually performed**				**protocol** ***p*** **-value**
Intensity (%1RM)	69.9 ± 2.7	68.6 ± 3.5	68.2 ± 2.5	0.16
Load (kg)	45.3 ± 10.1	44.1 ± 8.9	45.1 ± 8.8	0.26
MPV-best (m·s^−1^)	0.56 ± 0.05	0.58 ± 0.06	0.58 ± 0.05	0.29
MPV-average (m·s^−1^)	0.53 ± 0.04	0.51 ± 0.05	0.51 ± 0.05	0.35
MPV-last (m·s^−1^)	0.50 ± 0.05	0.52 ± 0.07	0.51 ± 0.06	0.54
VL (%)	11.0 ± 6.1	10.3 ± 7.4	13.5 ± 6.5	0.44

Data are mean ± standard deviation, (*n* = 14); LOW: the low-volume protocol; MOD: the moderate-volume protocol; HIG: the high-volume protocol; %1RM: percentage of one-repetition maximum; Load: absolute load lifted; MPV: mean propulsive velocity; best: the fastest MPV during the protocol (without including warm-up); average: average MPV during the protocol (without including warm-up); last: MPV of the last repetition; VL: magnitude of velocity loss expressed as relative difference between the best and the last MPV.

**Table 2:** The percentage of repetitions performed in each velocity range during each protocol.

Protocols	0.7-0.6 m·s^−1^ (% Reps)	0.6-0.5 m·s^−1^ (% Reps)	0.5-0.4 m·s^−1^ (% Reps)	0.4-0.3 m·s^−1^ (% Reps)
LOW	4.8 ± 12.1	64.3 ± 33.2	28.6 ± 28.2	2.3 ± 8.7
MOD	9.5 ± 19.2	47.6 ± 34.5	40.0 ± 37.1	2.9 ± 7.3
HIG	8.7 ± 17.6	44.9 ± 28.3	44.3 ± 33.5	2.1± 5.8

Data are mean ± standard deviation, *n* = 14. LOW: the low-volume protocol; MOD: the moderate-volume protocol; HIG: the high-volume protocol. % Rep: percentage of repetitions regarding the total repetitions performed in each protocol.

## Data Availability

The data shown in this study are available on request from the corresponding author. The data are not publicly available due to containing information that could compromise the privacy of research participants.
